# Common crossroads in diabetes management

**DOI:** 10.1186/1750-4732-2-4

**Published:** 2008-02-15

**Authors:** Michael Valitutto

**Affiliations:** 1 Borgess Center for Diabetes Care, Kalamazoo, Michigan, USA

## Abstract

The prevalence and impact of type 2 diabetes are reaching epidemic proportions in the United States. Data suggest that effective management can reduce the risk for both microvascular and macrovascular complications of diabetes. In treating patients with diabetes, physicians must be prepared not only to tailor the initial treatment to the individual and his or her disease severity but also to advance treatment as necessary and in step with disease progression.

The majority of patients with diabetes are not at goal for glycated hemoglobin A1C, fasting plasma glucose, or postprandial plasma glucose levels. Although lifestyle changes based on improved diet and exercise practices are basic elements of therapy at every stage, pharmacologic therapy is usually necessary to achieve and maintain glycemic control. Oral antidiabetic agents may be effective early in the disease but, eventually, they are unable to compensate as the disease progresses. For patients unable to achieve glycemic control on 2 oral agents, current guidelines strongly urge clinicians to consider the initiation of insulin as opposed to adding a third oral agent. Recent research suggests that earlier initiation of insulin is more physiologic and may be more effective in preventing complications of diabetes. Newer, longer-lasting insulin analogs and the use of simplified treatment plans may overcome psychological resistance to insulin on the part of physicians and patients.

This article summarizes the risks associated with uncontrolled fasting and postprandial hyperglycemia, briefly reviews the various treatment options currently available for type 2 diabetes, presents case vignettes to illustrate crossroads encountered when advancing treatment, and offers guidance to the osteopathic physician on the selection of appropriate treatments for the management of type 2 diabetes.

## Background

The prevalence of type 2 diabetes is reaching epidemic proportions in the United States. Impacting an estimated 20.8 million people – 7% of the overall US population, including an astounding 21% of those older than 60 years [1] – this progressive disease can lead to macrovascular and microvascular complications [2]. Macrovascular complications (cardiovascular disease and stroke) account for approximately 65% of all mortality in people with diabetes. Diabetes also is the leading cause of kidney failure and new cases of blindness among adults aged 20–74 years [2]. Other microvascular complications of diabetes include periodontal disease resulting in tooth loss, neuropathy leading to limb amputation, and numerous other potentially life-threatening complications [2].

Type 2 diabetes has a significant effect on quality of life. Patients are often concerned about disease progression or complications, social stigma, convenience/impact on lifestyle, and cost. Many have misperceptions regarding the complexity of treatment regimens [3,4]. These psychosocial issues also can impede glycemic control. Treatment must be tailored to the severity of disease. The start of insulin therapy is often delayed unnecessarily because both patients and practioners may underestimate the importance of glycemic control. [5].

The array of treatment options can be complex and challenging. Several newer therapeutic options have become available within the past decade. Physicians designing a treatment strategy are faced with several decisions:

◦ Choice of the best therapeutic option with each step of advancing disease;

◦ When to advance therapy;

◦ When to initiate insulin; and

◦ The utility and place of newer agents in a treatment strategy.

Because primary care physicians provide diabetes care for the great majority of patients with type 2 diabetes (82%), it is important for this group of physicians to understand the risks associated with poor glycemic control and the strategies that will help more patients reach their glycemic targets [6]. The purpose of this article is to offer guidance to the osteopath on the selection of appropriate treatments for the management of type 2 diabetes.

### Overview: Disease pathology and treatment goals

**Figure 1:**
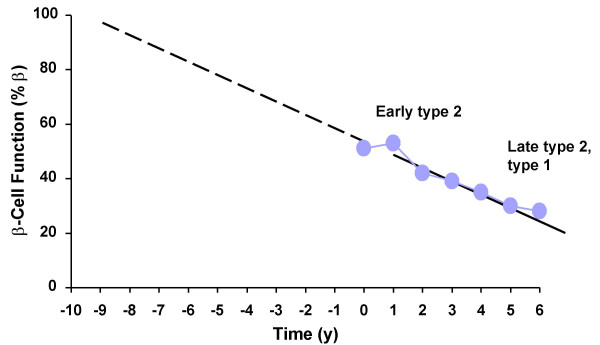
**Progressive loss of β-cell function in type 2 diabetes.** At diagnosis, a patient with type 2 diabetes has half the β cells as a person without type 2 diabetes. U.K. Prospective Diabetes Study Group. *Diabetes*. 1995;44:1249–1258 [8].

Type 2 diabetes is a progressive disease characterized by defects in insulin secretion and utilization [7]. In the normal postabsorptive state, glucose utilization in insulin-dependent tissue (primarily muscle) is balanced by glucose production [7]. In type 2 diabetes, tissues become insulin resistant, the ability of the pancreatic β cells to secrete insulin is impaired [7], and β-cell function declines progressively over time (Figure 1) [8].

The American Diabetes Association (ADA) and the American Association of Clinical Endocrinologists (AACE) have issued recommendations for target levels of glycemic control (Table 1) [9,10]. The ADA recommends that glycated hemoglobin A1C (hereafter A1C) be measured routinely in all patients with diabetes, initially to assess glycemic control and then as part of continuing care [9]. While A1C reflects the average level of glycemic control over a 3-month period of time, day-to-day control is demonstrated by both basal and postprandial plasma glucose (PPG) levels. Effective treatment, therefore, should encompass the means to control both basal and PPG excursions.

**Table 1:** ADA and AACE Glycemic Goals

	**ADA**	**AACE**
**A1C**	< 7.0%*	< 6.5%
**FPG**	90–130 mg/dL	< 110 mg/dL
**PPG**	< 180 mg/dL	< 140 mg/dL

A1C = glycated hemoglobin A1C; AACE = American Association of Clinical Endocrinologists; ADA = American Diabetes Association; FPG = fasting plasma glucose; PPG = postprandial plasma glucose.

*Physicians and patients should attempt to achieve a level as close to normoglycemia (< 6.0%) as possible without experiencing serious adverse events.

The role of postprandial versus basal hyperglycemia has been examined to ascertain the relative contribution of each to overall metabolic disequilibrium in type 2 diabetes [11]. In patients with mild to moderate hyperglycemia (A1C ≤ 8.4%), postprandial hyperglycemia has been shown to be an important contributor to the overall level of hyperglycemia [11]. Basal hyperglycemia becomes a major contributor in patients with poorly controlled and more extensive hyperglycemia (A1C ≥ 8.5%) [11]. However, these cutoff points are fluid; the respective contribution of each type of hyperglycemia shifts with the level of overall glycemic control as reflected by the A1C measure. The role of PPG elevations decreases as the patient's glycemic control deteriorates [11].

Improved glycemic control reduces the risk of microvascular complications and macrovascular events in type 2 diabetes [12,13]. Tight control in patients with type 1 diabetes also reduces the risk of macrovascular events and cardiovascular disease [14,15]. However, most patients with diabetes are not at goal for glycemic control or cardiovascular risk factors. According to recent data from the National Health and Nutrition Examination Survey, < 50% of patients with self-reported diabetes were at target A1C; 36% met target low-density lipoprotein (LDL) cholesterol goals for low-risk status; < 28% were at the low-risk status because of their high-density lipoprotein cholesterol levels; and < 40% met target blood pressure goals [16]. Long-standing failure to achieve glycemic control has been associated with increased risk for cardiovascular events [17]. The Heart Outcomes Prevention Evaluation study reported a progressive association between glycemia indices and incident cardiovascular events, nephropathy, and total mortality [17]. Therefore, an improvement in the achievement of these goals may greatly improve the health outcomes of patients with diabetes [9].

### Progressive therapeutic management of type 2 diabetes

**Figure 2:**
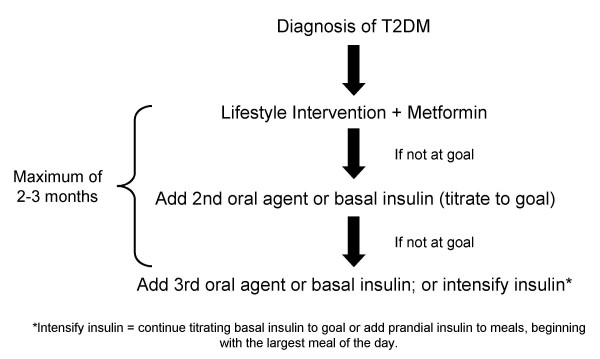
**Progression of therapy in type 2 diabetes. **ADA recommended algorithm. Nathan DM et al. *Diabetes Care*. 2006;29:1963–1972 [19].

Lifestyle modification is an important component of care throughout all stages of diabetes, and should be initiated and maintained as the basis of therapy (Figure 2) [18,19]. Lifestyle changes include medical nutrition therapy, physical fitness programs with 30 minutes of exercise 5 times per week, and weight loss (if the patient is overweight; 5%–7% reduction in body weight) [18]. These strategies have been shown effective in delaying the onset of type 2 diabetes in clinical studies [9,18] and decreasing A1C by 1.0% to 2.0% [19]. It is important to note that lifestyle modification is never abandoned in favor of medical therapy. Rather, it constitutes the backbone of diabetes therapy throughout disease progression [18,19]. Such lifestyle changes may increase the probability that other therapies will be effective [20].

As most patients will not achieve glycemic goals with diet and exercise alone, the ADA and the European Society for the Study of Diabetes suggest lifestyle intervention plus metformin as a first step in therapy [9]. Metformin is the preferred oral antidiabetic drug (OAD) if the patient is overweight, since it promotes weight loss and does not cause hypoglycemia [20]. If glycemic goals are not met with one OAD, a second is added; commonly, a sulfonylurea is added to metformin therapy [20]. As the disease progresses, treatment with additional medications becomes necessary to meet glycemic goals: a third agent from a different class, such as a thiazolidinedione, could be added or a basal insulin could be initiated [20]. A consensus statement from the ADA provides a comprehensive review of the benefits and disadvantages of OADs [19].

The decision of when to initiate insulin therapy can be challenging. Patients and physicians may resist this step in the mistaken belief that regimens are too complex and in the fear that hypoglycemia is difficult to avoid. However, most patients with type 2 diabetes will eventually require treatment with insulin due to the progressive decline of β-cell function (Figure 1), insulin resistance, and glucotoxicity that occur with the disease [7]. Many patient misconceptions and fears about insulin can be overcome with appropriate education and open communication. Furthermore, the use of newer, simple patient-driven algorithms permits patients to titrate insulin to goal with a reduced risk of hypoglycemia [21,22].

Currently available products offer opportunities for more individualized treatment and simpler regimens, and it is becoming increasingly clear that earlier insulin therapy can provide clinical benefit [23]. For example, systematically titrating bedtime basal insulin that had been added to oral therapy was shown to safely achieve an A1C of 7.0% in a majority of patients with type 2 diabetes whose A1C had been 7.5% to 10.0% while they were receiving oral agents alone [23].

Patients and clinicians are advised to avoid complacency and must aggressively move up the treatment ladder when glycemic targets are not met. Clinicians' failure to intensify therapy when indicated – so-called clinical inertia – may prevent patients from meeting their glycemic goals [24]. In one retrospective analysis, a quality improvement intervention aimed at overcoming clinical inertia in a diabetes clinic setting led to more frequent intensification of therapy and lower A1C levels [24,25].

#### Insulin: Benefits of early initiation

The long-standing assumption that insulin should be used as a last-resort intervention during treatment of patients with type 2 diabetes is now being challenged. Until recently, it was believed that insulin should be initiated only when combined lifestyle approaches and 1 or more OADs failed to achieve glycemic goals [26]. In addition to patients' fear of injections and concerns about lifestyle disruption, many physicians believed that insulin increased the risk of cardiovascular disease. On the contrary, studies have demonstrated that insulin has several potentially beneficial cardiovascular properties [26]. Among these is a marked reduction in inflammatory indices [27]. These benefits may not extrapolate to non-insulin antidiabetes agents, as recent data indicate a potential risk for cardiovascular morbidity and mortality in patients taking an oral thiazolidinedione agent [28], although these data must be interpreted with caution [29].

Data show that glucose is proinflammatory and that hyperglycemia is potentially toxic [30]. Insulin is an effective anti-inflammatory measure, since it normalizes plasma glucose and exerts significant anti-inflammatory effects [27]. Thus, the decision to delay insulin and instead add a third OAD may prolong the state of toxic hyperglycemia.

While the addition of basal insulin when OAD fails is now an established practice, whether to add insulin earlier or later in disease progression has remained controversial [31]. The United Kingdom Prospective Diabetes Study demonstrated the advantages of the early addition of insulin to conventional diet-plus-OAD treatment if the fasting plasma glucose (FPG) remained > 108 mg/dL despite maximal doses of sulfonylurea [31]. Over 6 years, ~ 53% of patients receiving sulfonylureas met the study requirement for additional insulin therapy [31]. Compared with the insulin-alone treatment group, significantly more patients in the added-insulin group had A1C < 7.0% over the 6-year study period, and their median A1C was significantly lower (6.6%) than that of the group taking insulin alone (7.1%) [31].

#### Basal and prandial insulin

**Figure 3:**
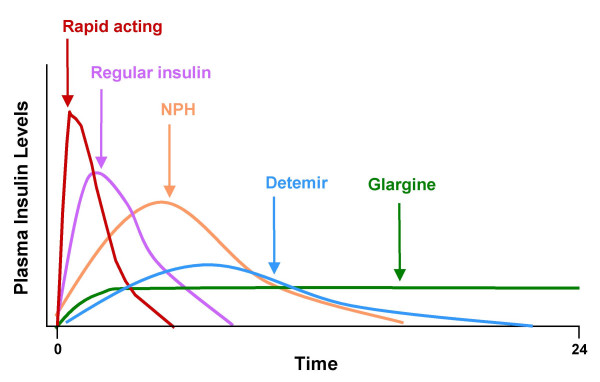
Idealized profiles of human insulin and analogs.

Table 2 lists currently available insulin formulations. Since the beginning of insulin therapy more than 75 years ago, a variety of improvements have resulted in the availability of numerous human insulin formulations with different onsets of action, peak concentrations, and durations of effect [32]. Judicious selection of specific insulin formulations to supplement OAD agents is an effective tactic to help patients reach glycemic goals [32] (Figure 3).

**Table 2:** Currently Available Insulin Formulations

**Insulin Formulation**	**Coverage**	**Duration of Action**	**Dosing**	**Special Considerations**
NPH insulin [69]	Basal	13 hours	Twice daily	Nocturnal hypoglycemia; morning hyperglycemia; intersubject variability
Insulin glargine [69,70]	Basal	24 hours	Once daily	Less risk of hypoglycemia (overall and nocturnal) compared with NPH insulin; once-daily dosing
Insulin detemir [38,45,71]	Basal	14 hours	Once or twice daily	Less nocturnal hypoglycemia and less weight gain compared with NPH insulin; most patients require twice-daily dosing
RHI [40]	Prandial	6–8 hours	30 minutes premeal	Limited mealtime flexibility
Insulin lispro [42,72]	Prandial	3–4 hours	Up to 15 minutes premeal or immediately postmeal	Pregnancy category B rating
Insulin aspart [40,44]	Prandial	3–4 hours	Up to 15 minutes premeal or immediately postmeal	Pregnancy category B rating.
Insulin glulisine [72,73]	Prandial	3–4 hours	Up to 15 minutes premeal or up to 20 minutes after start of meal	Only rapid-acting agent evaluated in conjunction with a dosing algorithm
**Premix (human)** 70% NPH, 30% RHI 50% NPH, 50% RHI	Basal-prandial	16–24 hours	Prebreakfast and presupper	Short- and long-acting components in fixed ratio; difficult to titrate, increased risk of hypoglycemia
**Premix (insulin analogs)** 75% NPL, 25% lispro 50% NPL, 50% lispro 70% protamine aspart, 30% aspart	Basal-prandial	16–24 hours	Prebreakfast and presupper	Short- and long-acting components in fixed ratio; difficult to titrate, increased risk of hypoglycemia

NPH = neutral protamine Hagedorn; NPL = neutral protamine lispro; RHI = regular human insulin.

Effective treatment should target both basal and PPG levels. Fasting and postprandial glycemia both are abnormal in type 2 diabetes, and the basal-bolus insulin strategy most closely resembles normal physiologic patterns [32]. As a starting point, a single injection of basal insulin can be used to provide 24-hour control of glycemia in patients in whom oral agents no longer constitute adequate therapy [32].

#### Basal insulin

Newer long-acting insulin formulations represent an improvement over neutral protamine Hagedorn (NPH) insulin, the basal insulin that has been used for many years. Recombinant DNA technology has produced insulin analogs – insulin glargine and insulin detemir – with structural modifications that improve their time-action profiles such that they more closely resemble physiologic insulin patterns [33].

Trials evaluating insulin glargine versus NPH insulin added to existing OAD therapy have found that treatment with either agent resulted in similar improvements in glycemic control [23,34,35]. However, insulin glargine was associated with less nocturnal hypoglycemia than NPH insulin [23,34,35]. This is likely due to the 24-hour duration of action with no pronounced peak activity of insulin glargine. Yki-Jarvinen et al performed a meta-regression analysis of clinical trials that used either NPH or insulin glargine as treatment options and observed that at equivalent rates of hypoglycemia, insulin glargine was associated with significantly lower A1C levels than NPH insulin [36].

Insulin detemir is approved for once- or twice-daily dosing and exhibits an activity peak at approximately 6–8 hours following administration [37]. Clinical trials comparing insulin detemir and NPH insulin have indicated that treatment with insulin detemir results in comparable improvements in A1C, less weight gain, and reduced risk for hypoglycemia [38].

### Clinical case: A patient recently diagnosed with type 2 diabetes

#### Case History

Mr. Madera is a 45-year-old Hispanic man recently diagnosed with type 2 diabetes. He is 5 ft 9 in tall and weighs 225 lb; his body mass index (BMI) is 33 kg/m^2^. He smokes 1 pack of cigarettes per day. During a recent visit to the clinic, his FPG level was 235 mg/dL and his A1C level was 10.6%. His blood pressure, total cholesterol, LDL cholesterol, and triglycerides were elevated. Mr. Madera's physician created a treatment plan that included patient education, diet, exercise, and smoking cessation. Additionally, oral therapy with metformin was initiated, along with aggressive management of the patient's cardiovascular risk factors.

At the 3-month follow-up, his A1C decreased to 9.2%. At that time, he was receiving 1000 mg of metformin per day. As the consensus algorithm from the ADA and the European Association for the Study of Diabetes recommends rapid intensification of therapy if patients fail to meet their glycemic goal with lifestyle changes in addition to treatment with metformin and if A1C levels remain > 8.5% [19], Mr. Madera's physician was concerned about his hyperglycemia and recommended the initiation of basal insulin therapy rather than the addition of a second OAD. However, Mr. Madera was concerned about injections and indicated that he preferred to delay the addition of insulin and wanted to try combination OAD therapy. Therefore, glimepiride was added to his current oral regimen at 4 mg once daily.

At his 6-month follow-up, Mr. Madera's A1C was 8.0%, and he had gained 4 lb. The physician discussed possible treatment options with Mr. Madera. His physician suggested initiating insulin therapy and demonstrated how to administer injections. Mr. Madera practiced the injection technique and decided to try treatment with insulin. Insulin glargine was added to Mr. Madera's existing regimen of metformin (1000 mg/d) and glimepiride (4 mg/d). Mr. Madera was instructed to begin with 10 units of insulin glargine administered at bedtime and adjust his dose weekly until he reaches a target FPG ≤ 100 mg/dL

Three months later, Mr. Madera was on 1000 mg/d of metformin, 4 mg/d of glimepiride, and 40U of insulin glargine administered once in the evening. His A1C had decreased to 7.2%, his FPG was 110 mg/dL, and he had no confirmed episodes of hypoglycemia. Mr. Madera reported that the insulin injections were not painful, and he will continue to titrate his dose of insulin glargine until his blood glucose reaches target. His weight had increased slightly, so the importance of continuing lifestyle modification was reinforced and a dietician was recommended.

#### Case discussion

This case highlights the need for aggressive management to achieve glycemic goals. Diet, exercise, and therapy with oral agents are all beneficial in patients with type 2 diabetes, but these interventions alone were unable to bring Mr. Madera's blood glucose levels to goal. At this point, some might consider adding a third oral agent. Nevertheless, clinical research has shown this approach to be less effective in glycemic control and less cost-effective compared with the initiation of insulin [39]. The addition of a basal insulin was able to bring Mr. Madera's blood glucose levels close to goal within 12 weeks, and with continued titration of his basal insulin component Mr. Madera should soon meet target glucose levels. It is important to stress that lifestyle modification must be continued throughout treatment.

#### Prandial insulin

When A1C levels are not at target, prandial insulin can be added gradually to basal insulin therapy to provide postprandial control of glycemia. Prior to the development of short-acting insulin analogs, prandial control with regular human insulin (RHI) often was challenging due to variability in absorption and time to reach peak levels, as well as to frequent hypoglycemia resulting from the use of higher insulin doses to compensate for postprandial hyperglycemia [40].

Compared with RHI, the 3 rapid-acting insulin analogs (insulin aspart, insulin lispro, and insulin glulisine) have absorption and action profiles that more closely resemble physiologic insulin secretion in response to a meal [41]. Insulin lispro has a more rapid onset, an earlier peak, and a shorter duration of glucose-lowering activity than RHI [42]. Ross et al reported better adherence with insulin lispro than with RHI in a study of patients with type 2 diabetes [43]. Lispro improved 2-hour PPG levels, quality of life, and overnight hypoglycemia rates, with A1C levels equivalent to those achieved with RHI. [43]. Insulin lispro is approved for use 15 minutes before or immediately after a meal [42].

Insulin aspart has a more rapid onset of action than RHI. However, the rate of insulin absorption and therefore the onset of aspart activity may vary by injection site, exercise, and other variables [44]. Aspart used with insulin detemir in basal-bolus therapy resulted in more predictable FPG levels and significantly lower within-person variation compared with a regimen of NPH plus RHI. Further, the risk of nocturnal hypoglycemia was 38% lower with the aspart plus detemir regimen [45].

Insulin glulisine added to insulin glargine produced effective basal-prandial glycemic control, even in obese patients. Interestingly, Jungmann et al have reported that postprandial insulin glulisine produced a slightly lower blood glucose profile than preprandial glulisine in obese patients who were also receiving basal insulin [46]. This may be advantageous for younger patients with type 2 diabetes, who often prefer postprandial injections. Bergenstal and colleagues demonstrated that the use of a novel mealtime dosing algorithm with insulin glulisine was a safe and effective alternative to carbohydrate counting [47]. In this study, patients used the algorithm to adjust doses of insulin glulisine by 1, 2, or 3 U based on premeal glucose patterns, with glargine as the bolus insulin [47]. Although this algorithm was specific to insulin glulisine, it may also apply to other rapid-acting insulin analogs; however, to date, there have been no studies assessing the use of this algorithm with other rapid insulins.

Although all 3 of these prandial insulin analogs have a rapid onset of action, slight differences exist in the approved timing of injections. Insulin lispro [42] is approved for use 15 minutes before or immediately after a meal; glulisine [48] for 15 minutes before or within 20 minutes after starting a meal; and insulin aspart is approved for administration immediately before a meal (start of meal within 5–10 minutes after injection) [44]. Irrespective of the specific insulin chosen, the insulin regimen should be customized to individual needs. Some patients may require 1 injection daily, while others may require 2–4. All of these prandial insulin analogs are available in disposable pen devices for easy administration.

#### Premixed insulin

While offering the convenience of 2 injections per day, premixed insulin poses a challenge with respect to dose titration, since the ratio of short-acting to long-acting components is fixed. Oikine et al have reported that twice-daily administration of premixed insulin resulted in improved glycemic control after morning and evening meals, but a change in overall A1C values was not observed [49]. In patients not reaching goal with twice-daily injections with premixed insulin, the addition of a prandial insulin injection at lunchtime can become necessary. In such cases, the morning insulin dose should be decreased accordingly [6]. Because it is important that patients adhere to a strict meal schedule to minimize the risk of hypoglycemia, treatment with a premixed insulin regimen can limit patient flexibility with respect to mealtimes.

### Clinical case: Recurrent hypoglycemia

#### Case history

Mrs. Martin, a 57-year-old woman, was diagnosed with type 2 diabetes 5 years prior and recently was referred for diabetes management. Her self-monitored blood glucose levels ranged from 250–350 mg/dL. She has a regular exercise routine – walking 4–5 days a week – and maintains a reasonable diet. This patient is 5 ft 7 in tall and weighs 170 lb, with a BMI of 26.6 kg/m^2^. She described burning pains in both feet at night. Her medications include glyburide 10 mg twice daily and metformin 500 mg twice daily; higher doses of metformin had caused diarrhea. Pioglitazone had been prescribed in the past, but its use was discontinued due to pedal edema. Her A1C level is 9.3%.

Mrs. Martin began treatment with basal insulin, and the dose was titrated to her FPG goal. However, her PPG remained elevated (high 200s). Her physician suggested supplementing with prandial insulin, but the patient was unwilling to take 4 injections per day. Basal insulin was discontinued and the physician was prescribed a premixed insulin regimen (eg, 70% basal insulin aspart protamine and 30% prandial insulin aspart with breakfast and dinner); the dose was titrated to 40 U twice daily.

Within a few weeks, Mrs. Martin returned to report erratic blood glucose levels (either in the high 200s or in the low 50s) and recurrent symptoms of hypoglycemia (shakiness, anxiety, sweating) in conjunction with low blood glucose measures. She was transitioned back to her previous basal insulin regimen, and a bolus regimen of insulin glulisine was initiated. Titration was completed in a simple stepwise fashion, starting with the largest meal and adding injections prior to each meal until target glycemic control (A1C < 7.0%, PPG < 140 mg/dL) was achieved. This regimen may afford the patient greater flexibility with fewer hypoglycemic episodes [47].

#### Case discussion

Hypoglycemia can be a bothersome, though rarely serious, adverse event in patients with type 2 diabetes who are treated with insulin therapy. This case illustrates how insulin therapy can be optimized to improve glycemic control and to avoid hypoglycemia. Patients often consider the initiation of insulin therapy a major barrier to overcome because of a misconception that regimens are complex. In this case, postprandial blood glucose was above the target range, thus requiring a prandial insulin to optimize glycemic control. Adding a fast-acting insulin analog to the basal insulin would have been effective, but the patient initially did not want to take 4 injections, so an analog mix was prescribed. This patient's blood glucose control then became erratic, and she experienced a number of hypoglycemic episodes. Although not all patients have the same experience, for this patient, multiple daily injections provided a physiologic approach to glycemic control and allowed the patient greater flexibility as the medication could be tailored to address her fluctuations.

### Newer agents

Among the most recent introductions to the market for antidiabetes agents are a new, inhaled prandial insulin [50] and several mimetic agents based on gastrointestinal peptide hormones that, in concert with insulin and glucagon, regulate fuel homeostasis and eating behavior [51]. Research to date shows promise for the noninsulin agents as adjuncts to basal and prandial insulin therapy, but their place in the diabetes treatment algorithm has yet to be determined.

#### Inhaled insulin

In January 2006 the US Food and Drug Administration (FDA) approved an inhaled powder form of recombinant human insulin for the prandial treatment of adult patients with type 1 and type 2 diabetes [52]. It is absorbed as quickly as subcutaneously administered rapid-acting insulin analogs and more quickly than subcutaneous RHI, with absorption independent of BMI [50]. Inhaled insulin provides postprandial glycemic control when added to long-acting basal insulin, with a duration of action comparable to that of RHI [53]. Pulmonary function assessment is required prior to initiating therapy [50]. Although a non-injectable route of administration may appeal to some patients, the inhaler unit may be cumbersome. In fact, as of October 18, 2007, Pfizer Inc. halted production of its inhaled insulin powder due to lack of uptake of the product; however, there are other devices in development. There are currently no head-to-head data comparing inhaled insulin with rapid-acting insulin analogs.

#### Incretin mimetics

The first such agent in its class, exenatide is an incretin mimetic that mimics the glucagonlike peptide 1, a natural hormone secreted in response to the ingestion of food [54]. Approved by the FDA in 2005 as an adjunct therapy for type 2 diabetes, exenatide given as twice-daily injections enhances insulin release in response to elevated blood glucose levels, inhibits glucagon secretion after meals, slows the rate of gastric emptying (which reduces nutrient absorption into the bloodstream), and increases satiety thereby promoting reduced food intake [54]. In clinical studies, exenatide improved glycemic control and, over time, A1C in patients whose type 2 diabetes had been worsening with maximal doses of a sulfonylurea, with metformin, and with combined sulfonylurea and metformin treatment [55-57]. Overall, A1C levels declined significantly over the course of the studies, plateauing after 12 weeks of therapy [55-57]. In the patients with type 2 diabetes who did not reach normoglycemia on a combination therapy of sulfonylurea and metformin, adjunctive exenatide or insulin glargine each reduced A1C by 1.1%, with about 46% of patients on each therapy achieving A1C ≤ 7.0% [58]. Insulin glargine, however, reduced FPG to a greater extent, and a greater percentage of patients achieved FPG < 100 mg/dL [58]. More patients withdrew from exenatide treatment due to nausea or other gastrointestinal symptoms (18 vs 1 in the insulin glargine group) [58]. Nausea affected approximately half of patients on exenatide in the studies, especially at high doses [55-57]. Minimal dose titration, mild hypoglycemia, and weight loss may be advantages of exenatide [59]. The FDA recently reviewed 30 postmarketing reports of acute pancreatitis in patients taking exenatide and requested that information about acute pancreatitis be added to the product label. Patients should be instructed to seek prompt medical care if they experience unexplained persistent severe abdominal pain which may or may not be accompanied by vomiting [60]. Other limiting factors of exenatide may be cost, gastrointestinal side effects, and potential interaction with oral contraceptives and antibiotics [59,61].

#### Pramlintide

An analog of amylin, a pancreatic peptide secreted by β cells in response to meals, pramlintide used in insulin-treated patients with type 1 diabetes and type 2 diabetes reduces PPG excursions, lowers A1C levels another 0.4% to 0.6%, increases satiety, and decreases food intake [62]. Its mechanism of action is independent of and additive to insulin. Approved in 2005, pramlintide is initiated at 15 μg subcutaneously with each meal and is titrated up to 30 or 60 μg per meal, depending on the dose-limiting side effect of nausea. Then the insulin dose is decreased by 50% or to the degree that maintains glycemic control without causing hypoglycemia [62]. Weight loss has been noted in obese and overweight patients [62].

#### Sitagliptin

Approved in 2006, sitagliptin inhibits dipeptidyl peptidase 4 (DPP-4), an inactivator of gut incretin hormones, which reduce postprandial and fasting glucose concentrations. By blocking this inactivation, the DPP-4 inhibitor increases active incretin levels and effects [63]. Sitagliptin 100 mg, given orally once per day, is approved for monotherapy or adjunctive therapy with metformin or a thiazolidinedione, or with the combination of sulfonylurea and metformin in patients with type 2 diabetes who have experienced inadequate glycemic response on OADs [64]. Clinical studies have shown improvements in A1C, FPG, and PPG with no increased risk of hypoglycemia and a slight increase in abdominal discomfort compared with placebo [63,65,66]. In March 2007, the FDA approved a combination product containing sitagliptin and metformin [67].

## Conclusion

Type 2 diabetes is a highly prevalent, progressive disease, characterized by defects in insulin production and utilization and is associated with significant morbidity and mortality.

The glucose levels of most type 2 diabetic patients are not well controlled. Therefore, treatment regimens should be individualized and optimized to achieve glycemic targets in addition to managing other risk factors.

Diet, exercise and oral agents are effective for reducing A1C; however, they do not sufficiently compensate for progressively worsening β-cell function and insulin resistance. Physiologic insulin replacement in the form of basal-bolus insulin manages background and postprandial glycemic excursions well. In addition, newer treatment options hold promise but require additional clinical study and evaluation to determine their appropriate place in a treatment paradigm.

## Competing interests

Dr. Valitutto has no competing interests to disclose.

## Authors' contributions

Dr. Valitutto was involved in the discussion of the concept of this article, directed the content of the initial outline, revised it critically for important intellectual content, and provided final approval of the manuscript.
